# Evidence for Integration of Cognitive, Affective, and Autonomic Influences During the Experience of Acute Pain in Healthy Human Volunteers

**DOI:** 10.3389/fnins.2022.884093

**Published:** 2022-05-26

**Authors:** Jocelyn M. Powers, Gabriela Ioachim, Patrick W. Stroman

**Affiliations:** ^1^Stroman Lab, Centre for Neuroscience Studies, Queen’s University, Kingston, ON, Canada; ^2^Department of Biomedical and Molecular Sciences, Queen’s University, Kingston, ON, Canada; ^3^Department of Physics, Queen’s University, Kingston, ON, Canada

**Keywords:** functional MRI, human neuroimaging, pain, cognitive/affective pain modulation, network connectivity, structural equation modeling

## Abstract

Our psychological state greatly influences our perception of sensations and pain, both external and visceral, and is expected to contribute to individual pain sensitivity as well as chronic pain conditions. This investigation sought to examine the integration of cognitive and emotional communication across brainstem regions involved in pain modulation by comparing data from previous functional MRI studies of affective modulation of pain. Data were included from previous studies of music analgesia (Music), mood modulation of pain (Mood), and individual differences in pain (ID), totaling 43 healthy women and 8 healthy men. The Music and Mood studies were combined into an affective modulation group consisting of runs with music and positive-valenced emotional images plus concurrent presentation of pain, and a control group of runs with no-music, and neutral-valenced images with concurrent presentation of pain. The ID group was used as an independent control. Ratings of pain intensity were collected for each run and were analyzed in relation to the functional data. Differences in functional connectivity were identified across conditions in relation to emotional, autonomic, and pain processing in periods before, during and after periods of noxious stimulation. These differences may help to explain healthy pain processes and the cognitive and emotional appraisal of predictable noxious stimuli, in support of the Fields’ Decision Hypothesis. This study provides a baseline for current and future investigation of expanded neural networks, particularly within higher limbic and cortical structures. The results obtained by combining data across studies with different methods of pain modulation provide further evidence of the neural signaling underlying the complex nature of pain.

## Introduction

Pain is a multidimensional experience which involves the integration of sensory, affective, cognitive, and autonomic features and, due to this complex nature, it is still poorly understood despite thousands of years of documentation (Dallenbach, 1939; Melzack and Wall, 1965; Millan, 2002; Raja et al., 2020). Although unpleasant in nature, the aversive qualities of pain function to encourage learning and memory in order to avoid future harm and increase evolutionary survival (Porreca and Navratilova, 2017). Functional magnetic resonance imaging (fMRI) is one of very few methods available to study the neural basis of the pain experience in humans, particularly because they can report their cognitive and emotional state along with ratings of their pain experience, unlike animal models. Although indirect, functional neuroimaging reaches beyond the valuable insights from behavioral studies of pain to record neural function via blood oxygenation-level dependent (BOLD) signals, which carry important temporal characteristics. Many fMRI studies have characterized neural responses to noxious stimuli in states of distraction and focus (attention), mood manipulation (emotion), music presentation, pharmacological intervention, and in states of chronic pain (Villemure and Bushnell, 2009; Lee et al., 2013; Dobek et al., 2014; Leung and Stroman, 2016; Cheng et al., 2017; McIver et al., 2018; Kornelsen et al., 2019; Warren et al., 2019).

Efforts to understand the cognitive and emotional dimensions of pain have significantly grown over the last decade, as definitions of pain have evolved past the simplistic notion of nociception (Porreca and Navratilova, 2017). While some have described cognitive modulatory processes of attention and emotion as distinct effects (Godinho et al., 2006; Villemure and Bushnell, 2009), Craig has described pain itself as a “homeostatic emotion” that indicates a deviation from homeostasis requiring a shift in cognitive, emotional, autonomic, and sensory factors in order to restore homeostatic balance (Craig, 2003a,b). Others have also shown evidence for integration of these effects via cortico-mesolimbic pathways which include dopaminergic and opioidergic signaling through regions such as the anterior cingulate cortex (ACC), nucleus accumbens (NAc), and amygdala, to motivate us to avoid pain and feel reward and relief when the pain subsides (Price, 2000; Becerra et al., 2001; Porreca and Navratilova, 2017; Gandhi et al., 2020).

This integration has been shown across studies of emotional regulation in chronic pain states where maladaptive emotional regulation has been implicated in the development of chronic pain (Koechlin et al., 2018; Lutz et al., 2018). Emotional regulation requires cognitive, behavioral and psychophysiological responses, encompassing attention, reward, and autonomic processes when appraising a stressor such as pain (Gross and Thompson, 2007; Lutz et al., 2018). Emotions such as fear and anxiety have evolutionary roots in the autonomic “fight or flight” response and can enhance selective attention to pain (Janssen and Arntz, 1996; Stroman et al., 2021). Furthermore, Fields has suggested a “decision circuitry” which allows us to appraise the threat of pain through the reward pathways (Fields, 2006), which are also intimately linked with emotional regulation networks through the mesolimbic system (Porreca and Navratilova, 2017). It is through this mesolimbic circuit that pleasurable music has been found to decrease pain, as the experience of pleasure can activate endogenous opioid and dopaminergic signaling (Leknes and Tracey, 2008; Porreca and Navratilova, 2017; Laeng et al., 2021). Finally, cognitive integration in this network has been shown to provide relief from pain through cortico-mesolimbic interactions including subregions of the frontal cortex, ACC, amygdala, hypothalamus, NAc, periaqueductal gray matter (PAG), and rostral ventromedial medulla (RVM) (Becerra et al., 2001; Wager et al., 2004; Brodersen et al., 2012; Lee et al., 2013; Porreca and Navratilova, 2017). Our cognitive and emotional states are powerful modulators of our experience of pain and are therefore, essential elements in the examination of healthy and maladaptive pain states.

Based on these findings, we chose to investigate the potential integration of emotion, autonomic function, and arousal in extended subcortical descending networks in the brainstem. A large dataset was compiled from prior functional MRI studies of pain to increase statistical power and precision. Data from prior mood and music modulation studies were combined into an “emotional modulation” condition, to be compared with their respective controls and a separate study of individual differences. We analyzed a pre-determined small brainstem network for evidence of differences in connectivity across conditions in regions involved in pain, arousal, limbic, and autonomic functions. The regions modeled in the network include the thalamus, hypothalamus, PAG, parabrachial nucleus (PBN), locus coeruleus (LC), nucleus gigantocellularis (NGc), nucleus raphe magnus (NRM), and nucleus tractus solitarius (NTS). We hypothesized that emotional stimuli during the pain experience in the mood and music data would reveal greater integration of these effects, compared to control conditions using only noxious stimuli.

## Materials and Methods

### Participants

Functional MRI data spanning the brainstem and spinal cord were obtained from 51 healthy participants (8 male, 43 female) in three prior studies conducted in our lab. These studies included (1) music analgesia (“Music”) (Dobek et al., 2014), (2) effects of mood on pain perception (“Mood”) (McIver et al., 2018; Kornelsen et al., 2019), and (3) individual differences of pain [“Individual Differences (ID)” (Khan and Stroman, 2015), see Table 1 for study details]. Only details relevant to the current investigation will be outlined here, for additional information on each study, please see the published manuscripts. In order to compare the intervention conditions to the ID study as a neutral control, data from the Mood and Music studies were combined based on the cognitive and emotional effects present. Positive mood and music + pain conditions were combined into one group based on the shared positive emotional valences, while neutral mood valence and no-music conditions were combined into another condition which we used as a control. Efforts were taken in the Music study to ensure that the participant-selected music fulfilled criteria for positive emotional valence, and the positive, neutral, and negative valences were standardized in the Mood study based on images obtained from the International Affective Picture System (IAPS) database (Lang et al., 2008). The negative emotional valence was not used here as it lay outside of the scope of the current investigation. In total, we combined data to produce three study groups: “Individual Differences” as a neutral control, “Mood and Music,” and their “Control” conditions. All studies had been approved by the Queen’s University institutional ethics board for human research, and informed consent was acquired prior to participation.

**TABLE 1:** Study group demographics and details of stimulation paradigms.

Study group	N (M:F)	Age range (Mean)	Average pain rating (±*S.D*.)	Stimulus temp. (± *S.D*.)	Stimulus timing (pre–stim–post)	Study conditions
Individual differences (Khan and Stroman, 2015)	18 (8:10)	18–45 Lutz et al., 2018	42.0 ± 18.0	49°C for all participants	50 s–30 s–75 s	6 repeated runs of the pain paradigm
Music analgesia (Dobek et al., 2014)	12 (0:12)	18–40 Stroman et al., 2021	53 ± 2.8 (Music condition) 57 ± 2.7 (No-music condition)	48.3 ± 1.3	50 s–30 s–75 s	4 repeated runs in each music and No-music conditions, interleaved
Effects of mood (McIver et al., 2018; Kornelsen et al., 2019	21 (0:21)	18–30 Gross and Thompson, 2007	46.2 ± 13.1 (Positive condition) 48.5 ± 13.1 (Neutral condition) 48.8 ± 12.3 (Negative condition)	49.1 ± 0.8	50 s–30 s–75 s	4 repeated runs in each positive, negative, and neutral emotional valences, interleaved

### Participant Training

**FIGURE 1:**
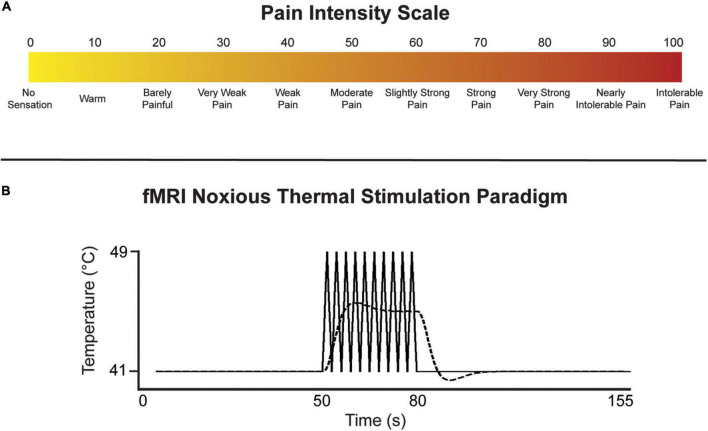
**(A)** Numerical pain intensity scale used to calibrate participants in the Mood and Music studies, and to allow participants to rate their level of pain to the thermal stimulus during each fMRI run in all studies. **(B)** Noxious thermal stimulation paradigm used for each study. The solid line represents the baseline temperature and the block-like noxious stimulation spikes in temperature over 8°C. The indicated temperatures were used in the ID study, while participants were individually calibrated in the Mood and Music studies. The dashed line represents the predicted BOLD response.

Prior to the imaging session in each of the studies, participants underwent a training session in which they were familiarized with study procedures, the noxious stimulus, and how to provide ratings of their perceived pain intensity on a 100-point scale (Figure 1A). These studies employed similar experimental thermal pain stimulation paradigms over multiple fMRI time-series acquisitions (“runs”). An MR-compatible Medoc^®^ Peltier thermode (Ramat Yishai, Israel) with a 3 cm square surface was applied to the thenar eminence of the right-hand palm in the Music and Mood studies (corresponding with the C6 dermatome), and to the right-hand palm under the fifth digit in the ID study (C8 dermatome). As the current investigation is focused on connectivity across specific regions in the brainstem, we do not believe that the location of afferent input to the spinal cord will significantly impact the results.

The stimulation paradigm was introduced to the participants during the training session, allowing them to anticipate the timing of baseline (i.e., warm, non-painful) and noxious stimulation periods. The stimulation paradigm was identical across all studies, consisting of an initial 50 s of baseline, followed by 30 s of stimulation, and another 75 s of baseline for a total of 2 min 35 s in each run (Figure 1B). While a noxious stimulation temperature of 49°C was used for all participants in the ID study, each participant in the Mood and Music studies was individually calibrated to elicit moderate pain (50 units on a numerical pain intensity scale, Figure 1A). The stimulus was held at a constant warm adaptation temperature during the baseline period (41°C in the ID study, and 8°C lower than the individual calibrated noxious temperatures in the Mood and Music studies) and ramped up by 8°C to the noxious temperature. During the 30 s of noxious stimulation, the thermode delivered a series of 10 heat spikes in a block-like paradigm in order to produce a sustained BOLD response. The stimulation temperature rapidly increased to the target/calibrated noxious temperature over 1.5 s followed by a rapid decrease to the baseline temperature over 1.5 s. After 30 s of noxious stimulation, the thermode temperature decreased to baseline for the remainder of the run. Participants were instructed to silently rate each noxious spike in temperature on the pain intensity scale and report their highest rating at the end of the practice run in the sham MRI, and subsequently each run in the imaging session.

### Functional MRI Data Acquisition

For the imaging session, each study employed identical imaging methods, while investigating different properties of the pain experience in healthy individuals (Table 1). The Individual Differences study acquired data in 6 runs for each participant, all of which employed the 2 min, 35 s noxious stimulation paradigm. The Mood study compared three conditions to elucidate the effects of different emotional valences on the experience of pain. Data were acquired in 4 runs of each positive, negative, and neutral emotional valences with simultaneous presentation of the noxious stimulation paradigm. Each emotional valence was presented using validated images from the International Affective Picture System (IAPS) database (Lang et al., 2008). For the purposes of this investigation, only the positive and neutral emotional valences were used to better compare with the Music study as none of the music selections elicited negative emotional responses. The Music study compared simultaneous presentation of pleasurable music (selected by each participant) with the noxious stimulation paradigm, with a No-Music condition of noxious stimulation alone. Four runs were collected in each condition for each participant. Participants were given at least 2 min of rest between runs in all studies in order to set up the next scan, and to avoid over-sensitizing nociceptors in the skin with repeated thermal stimulation.

Image data were acquired in the brainstem and spinal cord using a Siemens Magnetom Trio, 3 tesla, whole-body MRI system (Siemens, Erlangen, Germany). Initial localizer scans were acquired in three planes to assist subsequent slice positioning. In order to optimize spatial fidelity and blood oxygenation-level dependent (BOLD) sensitivity at the base of the skull and around the vertebrae, functional MRI data were acquired using a T_2_-weighted half-Fourier single-shot fast spin-echo sequence. While T_2_* weighting is typically used for functional imaging of the brain, T_2_-weighted BOLD acquisitions have been extensively validated and provide optimal image quality in the brainstem and spinal cord (Powers et al., 2018). Comparison studies have also demonstrated that the results obtained with the two methods are similar (Stroman et al., 2020). The 28 × 21 cm field of view spanned a 3D volume from the first thoracic vertebra to the superior edge of the thalamus. Nine contiguous sagittal slices were imaged with a repetition time (TR) or 6.75 sec/volume and an echo time (TE) of 76 ms, with 1.5 × 1.5 × 2 mm^3^ resolution.

### Data Analysis

#### Data Pre-processing

Functional imaging data were analyzed in MATLAB^®^ (MathWorks, Natick, MA), using custom-written software. Data were co-registered in 3D to correct for bulk motion using non-rigid registration (Myronenko and Song, 2010), and the required shifts were recorded (Muller et al., 2008). Images were subsequently resized to 1 mm cubic voxels and spatially normalized to an anatomical template of the brainstem and spinal cord generated in 3D using data from 356 people, as described previously (Bosma et al., 2015; Khan and Stroman, 2015). An anatomical atlas based on this template was used to define the boundaries of regions of interest (ROIs) in 3D; expected locations were compiled from and numerous other anatomical atlases and published papers (Lang and Bartram, 1982; Talairach and Tournoux, 1988; Lang, 1993; Gray et al., 1995; Naidich et al., 2009). The first two volumes of each time-series were removed to avoid variable T_1_-weighting, and to ensure data were analyzed only once a steady-state was reached. A general linear model (GLM) was also used to remove physiological noise and bulk motion effects using terms for bulk motion parameters, signal variations in white matter regions, and models of cardiac-related noise based on recordings of the peripheral pulse.

#### Structural Equation Modeling

**FIGURE 2:**
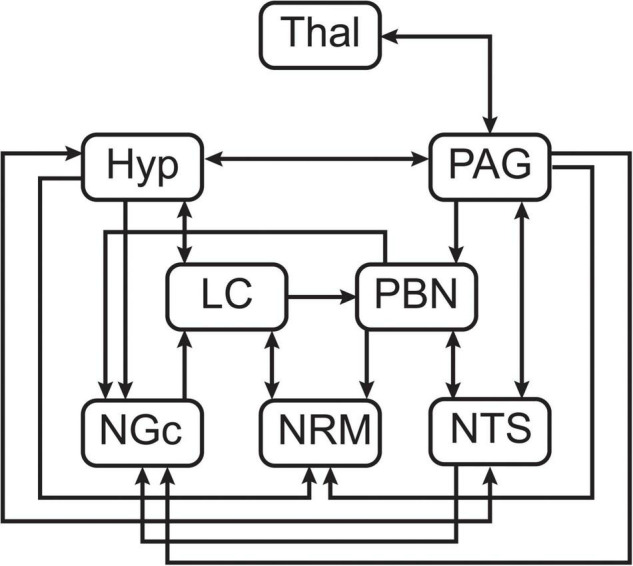
Pre-defined anatomical model of connections between brainstem regions of interest.

Following pre-processing, voxel data for ROIs were converted to the percent signal change from the time-series average, and each of the regions was divided into 5 sub-divisions using k-means clustering. The sub-regions were identified based on functional characteristics of the voxel time-series data to account for potential multiple functions in each region, and to group voxels with significant BOLD responses separately from non-responding voxels or those dominated by physiological noise. Structural equation modeling (SEM) is a data-driven method (i.e., the time-series responses are not modeled) which uses time-series BOLD responses averaged over the sub-regions to identify patterns of correlation and covariance across regions (Stroman, 2016; Warren et al., 2021). The goal of SEM is to explain as much of the variance in the BOLD signal as possible in a target region within a predefined neuroanatomical network (Kline, 2016; Stroman, 2016). The anatomical model is based on known connectivity between subcortical anatomical regions, including directionality, as described by Millan (2002) (Figure 2). The current investigation is focused on a small set of regions within a larger pain processing network used in previous studies in our lab (Dobek et al., 2014; Khan and Stroman, 2015; McIver et al., 2018). This small network is particularly focused on direct connectivity between pain-related regions and those with overlapping functions in emotion and autonomic processing in the brainstem, and those that have been implicated as downstream regions involved in cognitive modulation of pain. The regions included in this network are the thalamus (Thal), hypothalamus (Hyp), PAG, PBN, LC, NGc, NRM, and NTS.

Data were combined across runs from each participant in order to investigate variations in connectivity across individuals. The SEM analysis was applied by calculating linear weighting factors (β) for each target region by means of a GLM; these β represent the relative contribution of each input to a region. For example, if region A receives input signaling from regions B and C, and the BOLD time-series responses in these regions are S_A_, S_B_, and S_C_, respectively, then: *S*_*A*_ = β_*AB*_*S*_*B*_ + β_*AB*_*S*_*B*_ + *e*_*A*_, where *e*_A_ is the residual and the weighting factors (β) reflect the strength of connectivity between regions (Stroman, 2016). Each target region and its unique combination of sources is referred to as a “network component,” and networks were investigated for every combination of anatomical sub-regions in order to identify which sub-regions gave the best fits to the measured data. All time periods of interest were investigated (i.e., Before/Expectation, During/Stimulation, and After/Relief).

Significance of β-values (linear weighting factors for each connection) were determined against the null hypothesis (β = 0) based on the estimated standard error of β. Significance was inferred at a family-wise error corrected p_FWE_ < 0.05, accounting for the total number of network components that were tested across combinations of anatomical sub-regions. Connectivity weighting factors (β) were also analyzed across individuals by computing correlations of β-values with individual pain ratings. *R*^2^-values from these correlations were converted to Z-scores using the Fisher Z-transform with the number of participants. The significance was estimated based on a normal distribution and was inferred at a family-wise error corrected p_FWE_ < 0.05.

#### Analysis of Covariance

Analyses of covariance (ANCOVA) were applied to the β-values from SEM as a means of comparing the ID, Mood and Music, and Control conditions, in order to elucidate the relationship between the study condition and individual pain ratings across the three time periods of interest. Connectivity weighting factors (β) were used as the dependent variable, while the study condition was used as a discrete independent variable and pain ratings were used as a continuous independent variable for each time period (i.e., Group X Pain Rating, before, during, and after pain). Groups were compared in pairs in relation to connectivity weighting, pain rating and time period (e.g., ID vs. Mood and Music, Mood and Music vs. Control, Control vs. ID). This allowed for specific comparisons of the modulated conditions (Mood and Music) against a neutral control (ID), their own study controls (Neutral Mood and No-Music), and also for a comparison of ID against the Controls. The significance of the results was inferred at a false discovery rate (FDR) controlled p_FDR_ < 0.05.

## Results

### Structural Equation Modeling

Structural equation modeling analyses identified significant connections within the small brainstem network that was analyzed, across all conditions and time points of interest (Table 2). All conditions exhibited distinct patterns of connectivity in the expectation, noxious stimulation, and relief periods, however, only the ID condition revealed connections which significantly correlated with pain ratings across individuals. Significant connectivity in the ID condition was localized within four of the pre-defined regions of interest primarily involved in pain signaling and autonomic regulation. In the period before stimulation (expectation), connectivity in the ID condition is seen from the PAG to the NTS, however, during the experience of pain, only PAG→NRM connectivity is significant, indicating a change in function during stimulation. In the relief period after the noxious stimulation, the significant connectivity changes again to the PBN→NTS connection.

**TABLE 2:** Connectivity weighting factors (β), calculated by SEM, that are significantly different than zero (p_FWE_ < 0.05), bold values indicate correlation with pain ratings.

Condition	Source region	Target region	Before stimulation	During stimulation	After stimulation
Individual differences	PAG	NTS	**0.15 ± 0.05**	–	–
	PAG	NRM	–	0.55 ± 0.09	–
	PBN	NTS	–	–	**0.34 ± 0.07**
Mood and music	PBN	NRM	–0.29 ± 0.06	–	–
	Thalamus	PAG	0.62 ± 0.13	–	0.63 ± 0.13
	PAG	NRM	–	0.41 ± 0.09	–
	PBN	NTS	–	–	0.33 ± 0.06
Control	Hypothalamus	PAG	0.30 ± 0.05	–	–
	PAG	NTS	0.17 ± 0.03	–	0.25 ± 0.05
	PAG	Hypothalamus	0.14 ± 0.02	0.13 ± 0.03	–
	PBN	NTS	–	–	0.14 ± 0.03

SEM applied to the combined Mood and Music condition revealed descending connectivity from the PBN to the NRM and Thalamus to the PAG in the expectation period before noxious stimulation. During stimulation, the network shifted toward the more commonly described PAG→NRM modulatory descending pain signaling pathway. In the period following painful stimulation, signaling within the network returns to the Thalamus PAG connection, with added modulation from the PBN to the NTS. The Thalamus and PBN are both consistent regions involved in network connectivity in the periods before and after noxious stimulation.

The combined Control condition (Neutral Mood and No-Music) consisted largely of Hypothalamus and PAG involvement. Before noxious stimulation, SEM revealed reciprocal Hypothalamus **↔** PAG connectivity, as well as a descending projection from the PAG to the NTS. The ascending PAG→Hypothalamus connection was observed in isolation during the noxious stimulation period, while PAG connectivity returned toward the NTS in the period after stimulation along with a PBN→NTS connection. Notably, all connections before and during noxious stimulation included the PAG, while both connections after stimulation included the NTS. Additionally, in all three study conditions, the PBN connected with the NTS in the relief period after stimulation.

### Analyses of Covariance

**FIGURE 3:**
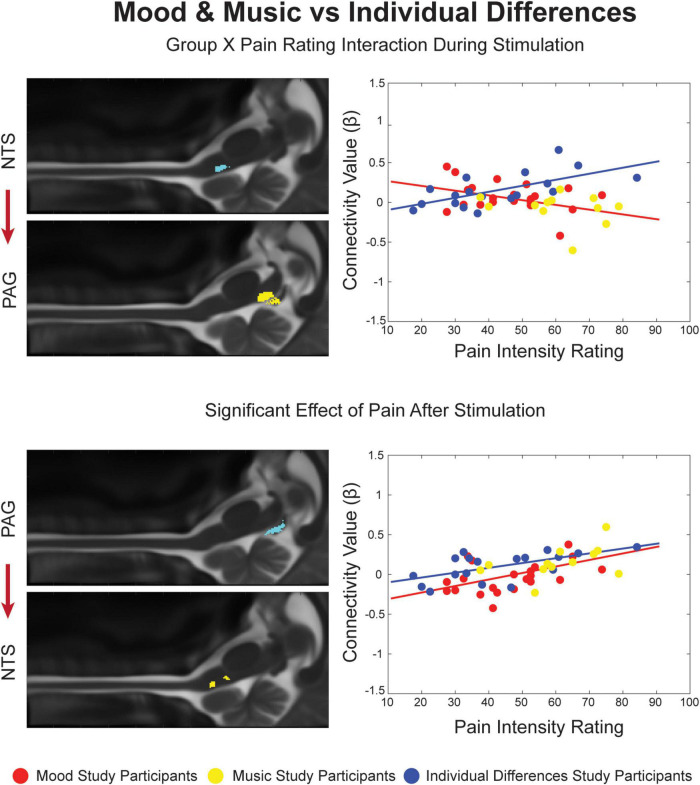
Significant Group X Pain Rating ANCOVA results for the Mood and Music condition and Individual Differences. Regions are highlighted and labeled on sagittal slices of the anatomy **(left)**. The figure legend refers to the plotted results of the ANCOVA **(right)** with red and yellow points representing Mood and Music study participants, respectively, corresponding to the red trendline as a combined group. The blue points and trendline represent the separate Individual Differences condition.

**FIGURE 4:**
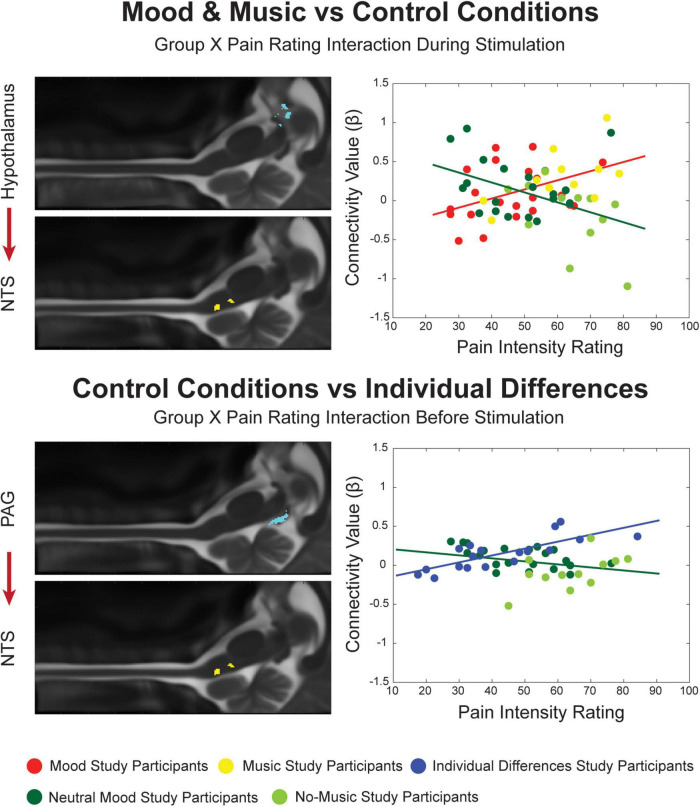
Significant Group X Pain Rating ANCOVA results for the Mood and Music condition, their combined Control conditions and Individual Differences. Regions are highlighted and labeled on sagittal slices of the anatomy **(left)**. The figure legend refers to the plotted results of the ANCOVA **(right)** with red and yellow points representing Mood and Music study participants, respectively, corresponding to the red trendline as a combined group. The blue points and trendline represent the separate Individual Differences condition. The Neutral Mood and No-Music conditions are represented as dark and light green, respectively, corresponding to the dark green trendline as the combined control condition.

All connections identified by SEM in at least one condition were applied toward analyses of covariance. The ANCOVA results revealed significant variation across brainstem connectivity in relation to each study condition (ID, Mood and Music, and Control) and individual pain ratings for each of these conditions in all three time periods of interest (Figures 3, 4). Four connections showed significant main effects of Group or Pain Ratings, or an Interaction between these variables. In the Mood and Music vs. ID comparison (Figure 3), an interaction effect was present during noxious stimulation in the ascending NTS→PAG connection. As pain ratings across individuals increased in the Mood and Music condition, connectivity weighting values (β) decreased, while the ID group showed a positive relationship between pain ratings and β. This group comparison also revealed a main effect of Pain Ratings in the period after stimulation in the reciprocal descending connection from the PAG to the NTS. A positive relationship between β-values and pain ratings was seen across both study groups.

When the Mood and Music groups were compared with their combined Control conditions, connectivity across groups was found to be significantly different dependent on the pain ratings of each individual (Figure 4). An interaction effect was seen during the experience of pain in a connection from the hypothalamus to the NTS. While β-values increased with pain ratings in the Mood and Music conditions, the opposite relationship was seen in the Control conditions. Lastly, an interaction effect was seen in the comparison of the ID group with the Control group, in the period before stimulation, in a descending connection between the PAG and NTS. While the ID group showed a positive relationship between β-values and pain ratings, the Control condition demonstrated a negative trend (Figure 4).

## Discussion

Our lab has compiled a large database of functional MRI studies of pain in healthy individuals and disease states across many years and different types of interventions. Due to the wealth of information contained in this database, and its research potential, new insights can be gained from mining this previously collected data. The current investigation sought to compare affective interventions during the experience of pain and contrast them with control conditions including a separate, neutral control study (ID). We hypothesized that, due to the involvement of cognitive and emotional processes and reactions within the Mood and Music studies, they could be combined into an affective modulation condition which would reveal patterns of network connectivity unique from the ID study and the combined Neutral Mood and No-Music controls, specifically involving integration across mesolimbic and autonomic regions. SEM analyses demonstrated significant connectivity across all study conditions and time periods of interest, while the ANCOVA revealed specific differences in connectivity across groups, reflecting individual differences in pain ratings and β-values. Our results support the hypothesis that mesolimbic and autonomic regions in the brainstem serve unique, but integrated roles in cognitive/emotional modulation of pain, and that network connectivity in Music and Mood group differs from control conditions. However, we also found interesting differences in connectivity across the control groups, and evidence of autonomic priming before the onset of pain, with additional autonomic involvement during and after noxious stimulation. These findings are in agreement with recently published investigations from our lab (Stroman et al., 2018, 2021; Warren et al., 2019, 2021; Ioachim et al., 2020).

Structural equation modeling revealed different patterns of connectivity within the small network model across each study condition. The ID condition involved connectivity from the PAG and PBN to the NTS both before and after stimulation, respectively, and both of these connections were correlated with individual pain ratings. In addition to pain processes, the PBN and NTS are implicated in arousal and autonomic regulation, which have been shown to play a role in anticipation of pain and have been shown to engage in the descending modulatory pathway at the offset of painful stimuli (Bingel et al., 2011; Stroman et al., 2018). Furthermore, regions involved in descending regulation of pain, emotion, and reward have been implicated in the expected relief from pain (Porreca and Navratilova, 2017; Stroman et al., 2018).

Interestingly, during pain, the network we analyzed seems to “default” to a classic descending modulatory pathway from the PAG to the RVM (which includes the NRM). The PAG→RVM spinal cord pathway has been described in great detail as the main route for descending regulation of pain since the PAG contains a high concentration of opiate neurons with efferent spinal connections (Melzack and Wall, 1965; Fields and Basbaum, 1994; Millan, 2002; Tracey et al., 2002). The combined Mood and Music condition also demonstrated this default PAG NRM pathway during the experience of pain, with some thalamic and autonomic influence in the periods before and after stimulation. Descending projections from the thalamus to PAG and from the PBN to the NRM and NTS are seen in these periods, lending to the experiences of anticipation and relief (Loggia et al., 2014). In 2001, through early fMRI studies, Becerra et al. (2001) found that signaling in reward pathways (including the VTA and NAc) correlated with activity in the PAG in an early period of pain anticipation, while the pattern of concurrent signaling reverted back to default pain circuitry in late anticipation of pain. Wager et al. (2004) have since suggested that the prefrontal cortex signals to opioidergic midbrain regions during the anticipation of pain in order to dampen the pain response, and although these regions may not directly be associated with attention, the expectation of pain may involve specific opioid signaling activation. This evidence lead to Fields’ “decision hypothesis” of pain anticipation where individuals must consider potential tissue damage in order for reward pathways to access early information regarding noxious stimuli, also suggesting that “reward” pathways should be renamed “decision circuitry” (Fields, 2006).

Similar to the ID condition, the combined Control condition (Neutral Mood and No-Music) included PAG→NTS connections before and after stimulation, in addition to PBN→NTS connectivity after stimulation. Once again, there is greater arousal and autonomic involvement outside of the stimulation period. Interestingly, outside of the affective modulation conditions, reciprocal connections between the PAG and hypothalamus were seen before and during stimulation. The hypothalamus is involved in limbic functions including the expression of emotions including aversion, pleasure and displeasure (Kropotov, 2016), however, it also contributes largely to the pain response through both sympathetic and parasympathetic pathways (Millan, 2002; Bernard, 2007). While this finding does not directly support the original hypothesis that limbic involvement would occur more in the affective conditions, this Control condition cannot be considered neutral as in the ID group since Neutral Mood pictures are still distracting and potentially modulatory compared to no other external stimuli being provided during the application of the noxious heat stimulus. Similarly, although the No-Music condition did not contain external stimuli in addition to noxious stimulation, the runs were randomly interleaved with Music runs, which may have had effects that carried over across runs (i.e., participants could have played/sung the music in their head during No-Music runs).

The analyses of covariance provided the opportunity to further investigate differences across study conditions, and periods of the stimulation paradigm, based on individual differences across participants, pain ratings, and connectivity weighting factors (β). The affective modulation conditions (Mood and Music) were compared with ID and the Neutral Mood and No-Music conditions, and the control conditions were compared with each other. In the Mood and Music vs. ID comparison, a Group X Pain Rating interaction was found during noxious stimulation in an ascending connection from the NTS to the PAG. This feedback may involve sending autonomic information regarding arousal due to stimulus presentation to “alert” the default pain modulation system through the PAG. Additionally, interaction effects provide important evidence for the precision and sensitivity of our methods as they show that we can detect connections which vary with individual differences in neural activity and pain sensitivity.

In this comparison we also found a main effect of pain ratings after stimulation in a feed-forward connection from the PAG to the NTS, which may convey information regarding communication of after-sensations such as warmth, tingling, or burning sensations after stimulation ended. The main effect of pain ratings could indicate that, regardless of condition, each individual’s pain sensitivity determines the way that they feel relief from pain. The role of the NTS in autonomic processing and interoception supports this hypothesis, as the body evaluates its state of homeostasis after the experience of pain (Millan, 2002; Craig, 2003a,b). Participants’ minds were free to wander during the baseline periods and they may have focused their coping strategies on appraising their relief that the pain has ended and the physical and emotional expense of the experience. Descending signaling from the PAG also supports the integration of cognitive and emotional regulation during the relief period regardless of condition, as the PAG is a key region for integration of pain-related behaviors and risk assessment, motivating us to avoid threatening stimuli (Millan, 2002; Craig, 2003a; Deng et al., 2016). Stimulation of the NTS has been shown to produce antinociception, which may involve opioid signaling from PAG enkephalin-producing cells, and reciprocal endomorphin signaling to the PAG from the NTS (Lewis et al., 1987; Millan, 2002; Lv et al., 2010). Furthermore, the experimental acute pain stimulus can be classified as an “escapable” stressor, which has been shown to activate the sympathetic nervous system to monitor for danger. The PAG has been implicated in this process as stimulation of different regions produces different coping strategies and types of analgesia (Coulombe et al., 2016). The relationship between the PAG and NTS could serve to alleviate the last of the after-sensations experienced and provide feedback between sensory and autonomic systems during a time for appraisal of the previous, and the current state of bodily homeostasis.

In the Mood and Music vs. Control comparison, there was a similar interaction effect during noxious stimulation, however, in a descending connection from the hypothalamus to the NTS. This connection reveals communication between limbic and autonomic networks which differs between conditions in relation to individual pain ratings. As pain is a complex sensory experience involving cognitive, affective, and interoceptive control that is dependent on individual pain history and memory, it is important for these systems to interact in order to create awareness of, and reactions to, pain in an individualized way (Melzack and Katz, 2013). Research in the field continually provides evidence of this multi-sensory and cognitive integration leading to the unique experience of pain (Becerra et al., 2001; Bornhovd et al., 2002; Villemure and Bushnell, 2002; Godinho et al., 2006; Leknes and Tracey, 2008; Porreca and Navratilova, 2017; Moayedi et al., 2018; Thompson and Neugebauer, 2019; Yuan et al., 2019; Ioachim et al., 2020; Tu et al., 2020; Stroman et al., 2021; Warren et al., 2021) ng been implicated in the experience of pain, and complex communication between these regions is just beginning to be investigated in humans. Future studies may focus on expanding these intricate network analyses to include the higher limbic regions such as the amygdala, and other cortical regions including sensory, frontal/executive, and memory areas.

Finally, in the comparison between the control conditions (ID, Neutral Mood and No-Music), a Group X Pain Rating interaction effect was found in the descending connection PAG→NTS in the period before stimulation. This is similar to the Mood and Music vs. ID comparison where a PAG→NTS connection differed significantly based on pain ratings in the period after stimulation. Again, this shows interesting communication between primary pain and autonomic networks in a way that the PAG could be priming autonomic arousal or antinociception through the NTS in anticipation of the impending noxious stimulation (Lewis et al., 1987; Millan, 2002; Lv et al., 2010; Stroman et al., 2018, 2021). However, the interaction effect for this connection is also dependent on the condition, in contrast to the main effect of pain in the Mood and Music and ID comparison. One might expect that the Control and ID conditions should not significantly differ in network connectivity, however, as previously mentioned, the Neutral Mood and No-Music conditions may not be considered neutral or independent controls due to cognitive interference of the interleaved affective modulatory conditions.

While this investigation uncovered interesting properties of pain processing within the brainstem, there were limitations of the methods which should be considered when appraising the results. First, the use of secondary data limited the available comparisons and presented challenges for interpretation of results. These include potential sex/gender differences as the Mood and Music studies collected data from only women, whereas the ID study included both men and women. Additionally, participants were recruited for the Mood and Music studies in the luteal phase of their menstrual cycles, whereas female participants in the ID study were not screened for timing of their menstrual cycle. This could produce a subtle effect in the results based on differential opioidergic effects on pain perception across the cycle (Dreher et al., 2007). Additionally, the ID study employed a constant noxious temperature for all participants which elicited a wider range of pain ratings, where the Mood and Music studies used carefully calibrated temperatures to elicit a smaller, more standardized range of pain ratings, around moderate pain. This has implications in the analyses of covariance as the results were dependent on pain ratings, however, our statistical methods employed strict thresholds, producing robust and reliable results. The ID study also provided stimulation to the 5th digit side of the palm, in contrast with the Mood and Music studies that stimulated on the thenar eminence below the 1st digit, producing afferent signaling to the C8 and C6 spinal segments, respectively. While this would cause significant differences in results in investigations at the level of the cervical spinal cord, we do not expect significant effects in the brainstem. In addition, as previously mentioned, the Neutral Mood and No-Music conditions may not be considered unbiased, independent control conditions due to the presentation of IAPS pictures and potential cognitive and emotional interference from the interleaved test conditions within each study. Finally, due to the nature of this investigation into a small brainstem network, many complex interactions between a diverse array of brainstem, spinal cord and cortical regions are missed. Therefore, we have provided evidence for a small, focused window of interactions within the brainstem during different pain conditions.

This investigation sought to examine the integration of cognitive and emotional communication across brainstem regions involved in pain modulation by comparing data from previous fMRI studies of emotional and music modulation of pain. We found differences across conditions in connections related to emotional, autonomic, and pain processing in periods before, during and after noxious stimulation. These differences may help to explain healthy pain processes and the cognitive and emotional appraisal of predictable noxious stimuli, in support of the Fields’ Decision Hypothesis. While this work is a baseline for current and future investigations of expanded neural networks, particularly within higher limbic and cortical structures, this study provides evidence for the immense value in the analysis of combined data sets in order to fully understand the complex nature of the pain experience.

## Data Availability Statement

The data analyzed in this study is subject to the following licenses/restrictions: the datasets used in this publication have been anonymized and the researchers retain confidential access and ownership. Requests to access these datasets should be directed to PS, stromanp@queensu.ca.

## Ethics Statement

The studies involving human participants were reviewed and approved by the Health Sciences and Affiliated Teaching Hospitals Research Ethics Board. The patients/participants provided their written informed consent to participate in this study.

## Author Contributions

PS and JP designed the study and carried out data analysis. All authors contributed to interpretation of the results and writing of the manuscript.

## Conflict of Interest

The authors declare that the research was conducted in the absence of any commercial or financial relationships that could be construed as a potential conflict of interest.

## Publisher’s Note

All claims expressed in this article are solely those of the authors and do not necessarily represent those of their affiliated organizations, or those of the publisher, the editors and the reviewers. Any product that may be evaluated in this article, or claim that may be made by its manufacturer, is not guaranteed or endorsed by the publisher.
